# Worldwide Review of Dental Hygienists’ Extended Scope of Practice in Radiology

**DOI:** 10.1016/j.identj.2021.05.010

**Published:** 2021-07-06

**Authors:** Meryam Bozia, Erwin Berkhout, Fridus van der Weijden, Dagmar Else Slot

**Affiliations:** a Department of Oral Radiology, Academic Centre for Dentistry Amsterdam, joint venture between the Faculty of Dentistry of the University of Amsterdam and Vrije Universiteit Amsterdam, Amsterdam, The Netherlands; b Department of Periodontology, Academic Centre for Dentistry Amsterdam, joint venture between the Faculty of Dentistry of the University of Amsterdam and Vrije Universiteit Amsterdam, Amsterdam, The Netherlands

**Keywords:** Dental hygienist, Questionnaire, Task redistribution, Competences, Law, Oral radiology

## Abstract

**Objectives:**

This study aimed to summarise the competencies and legal position of the dental hygienist (DH) regarding oral radiology through a worldwide review.

**Methods:**

A structured and peer-reviewed online questionnaire of 27 questions was developed. This was emailed to all DH associations that are members of the International Federation of Dental Hygienists (IFDH) or European Dental Hygienists Federation (EDHF). After obtaining the data, all responding associations were contacted to confirm that the data were summarised in the correct order and were asked to provide further clarification of answers if necessary. A descriptive analysis was performed to summarise the data.

**Results:**

The response rate was 84%, as 26 out of 31 countries completed the questionnaire. In 78% of the countries, the DH can legally take intraoral radiographs, but in 42% of the countries, the dentist first needs to provide a referral or indication for a radiograph. In 46% of the countries, the DH may not formulate a diagnosis based on a radiograph. In only 27% of the countries, the DH can independently own radiographic equipment.

**Conclusions:**

The required qualifications, skills, and scope of practice of the DH regarding oral radiology vary by country and, within some countries, even vary by state or province.

## Introduction

The oral health care domain has a substantial variety of professions, with the oral surgeon, dentist, and dental hygienist (DH) being the most prominent. The DH profession has evolved since its establishment and continues to do so.^1^ Several studies have been published regarding the scope of practice of DHs. The profession and its development are globally diverse.^2^, ^3^, ^4^ As stated in the 21-nation comparative study by Johnson on the international profiles of DHs from 1987 to 2006, work roles and relationships have been evolving from the dentist-predominant, DH-as-auxiliary model to a more collegial model that involves greater collaboration in patient care.^5^

The evolving DH profession in the Netherlands is mostly due to new legislation that extends the scope of practice by shifting and redistributing tasks.^1^ Recently, the Dutch Ministry of Health and Welfare announced a redistribution of specific oral health care tasks between the dentist and DH. This redistribution consists of granting, based on strict criteria, selected DHs independency for treating primary caries and cavities, administering local anaesthesia, and using ionising radiation for intra-oral radiology.^6^ The DH is increasingly becoming an independent dental care professional without the need for supervision by a dentist, as predicted by Johnson et al.^5^ However, it has also been suggested that the evolution of the dental hygiene profession and its influence in terms of health outcomes and other effects merit further investigation.^5^ Such an investigation has not been performed for more than a decade.

Although the use of ionising radiation is associated with health hazard safety issues, little is known about the awareness and subsequent related behaviour of DHs concerning this matter.^6^^,^^7^ The worldwide regulations for the DH profession in using ionising radiation are unknown. These tasks have traditionally been primarily within the dentist's scope of practice. For instance, a worldwide assessment of DH practice in oral radiology and on the topic of competencies and their legal position is currently lacking. Such a review could be helpful for future guidelines, continental alliances, national regulations, professional development, and policy making. In addition, it can help formulate common frameworks. Therefore, this study aimed to summarise the scope of practice of the DH regarding oral radiology through a worldwide review.

## Materials and Methods

### Study outline, guidelines, and ethics

This paper is part of the project “Worldwide Dental Hygienists Extended Scope of Practice.” This project aims to review the competencies and legal position of the DH profession from a global perspective. The study was ethically approved by the Institutional Review and Ethics Board of the Academic Centre for Dentistry Amsterdam (ACTA; reference code 201913). This manuscript was prepared according to the guidelines of the Strengthening the Reporting of Observational Studies in Epidemiology (STROBE)^8^ and Checklist for Reporting Results of Internet (CHERRIES).^9^

### Target group

All national dental hygiene associations that were members of the International Federation of Dental Hygienists (IFDH) or European Dental Hygiene Federation (EDHF) in 2018 were contacted as the target group. For an overview of the associations from the 31 countries that were approached, see Table 1.

**Table 1:** Respondents: IFDH and EDHF member countries.

	IFDH member	EDHF Member	Responded Quest.	Responded Valid.
**Australia**	√	NA	√	√
**Austria**	√	√	√	√
**Canada**	√	NA	√	√
**Czech Republic**	√	√	√	√
**Denmark**	√	√	√	√
**Finland**	√	√	√	√
**Germany**	√	√	-	NA
**Ireland**	√	√	√	√
**Israel**	√	√	√	√
**Italy**	√	√	√	√
**Japan**	√	NA	√	√
**Korea**	√	NA	-	NA
**Latvia**	√	NA	√	√
**Lithuania**	-	√	√	-
**Malta**	√	√	√	√
**Netherlands**	√	√	√	√
**Nepal**	√	NA	-	NA
**New Zealand**	√	NA	-	NA
**Norway**	√	√	√	√
**Poland**	-	√	√	-
**Portugal**	√	√	√	√
**Russia**	√	√	√	√
**Singapore**	√	NA	√	-
**Slovak Republic**	√	√	√	√
**South Africa**	√	NA	√	-
**Spain**	√	√	√	-
**Sweden**	√	√	√	√
**Switzerland**	√	√	√	√
**United Arab Emirates**	√	NA	-	NA
**UK**	√	√	√	-
**USA**	√	NA	√	√
**n**	29	20	26	20

NA, not applicable; IFDH, International Federation of Dental Hygienists; EDHF, European Dental Hygienists Federation.

√ = yes.

- = no.

### Questionnaire development

An online questionnaire was developed to gather the data of interest. The research team conducted a scoping exercise and pilot study. The questionnaire was completed and reviewed by 5 dental hygienists from different countries, and it was peer-reviewed by the IFDH, the EDHF, and the Dutch Dental Hygienist Association (Nederlandse Vereniging van Mondhygiënisten, NVM) to ensure its comprehensibility and usability. This pilot study aided to rephrase the questions with commonly used and undisputed terminology. The 27-item online questionnaire included general questions regarding education, independent practice, task-delegating authority to auxiliary personnel, and indirect access to patients for dental hygiene care. Furthermore, the questionnaire included questions related to the legal status of tasks in the fields of oral radiology, cariology, and anaesthesia. The closed-ended questions in this questionnaire may limit the richness of the potential answers. This issue was overcome by adding an open-ended question at the end of each category. A combination of open-ended and closed-ended questions was used to allow responders to expand on their answers. The IFDH, EDHF, and NVM endorsed the final questionnaire.

### Procedure

The questionnaire was entered in Google Forms, a web-based data entry tool. Only those who received the link via email were able to open and complete the online questionnaire. The contact details for all national associations were obtained from the websites of the IFDH or the EDHF (Table 1). Participation was voluntary, and the target group was emailed in November of 2018 and informed of the purpose of the study. The email invitation included the link to the questionnaire, and a portable document format (PDF) file of that questionnaire. Therefore, respondents could prepare the questions before filling out the online form. In addition, information was given regarding the duration of the questionnaire, number of questions, the researchers, and how the data would be used and presented. The participants were also reassured that their contact details would remain anonymous.

A response time of 4 weeks was allowed, and follow-up was conducted through phone calls or via a reminder email to prompt associations that did not respond. One last reminder was sent to the nonresponding associations until the questionnaire was closed at the end of December 2018. The responses were automatically saved to a private database when the respondents completed the questionnaire. Every question required an answer before the next one could be addressed to ensure fully completed questionnaires. The extracted data were automatically entered in an Excel spreadsheet. All electronic data were stored at a safe location, and access requires a password.

After obtaining the data, all responding associations were contacted to validate that the data were summarised correctly by the research team and were asked to provide further clarification of the answers if needed. A descriptive analysis was performed to summarise the data.

## Results

Out of the 31 countries, 26 (84%) responded to the questionnaire (Figure). All of the returned questionnaires were completed and considered eligible. Two questionnaires were not entered through Google Forms but through a PDF that was sent directly by email to the research team. Germany, Korea, Nepal, New Zealand, and the United Arab Emirates did not respond.

**Figure:**
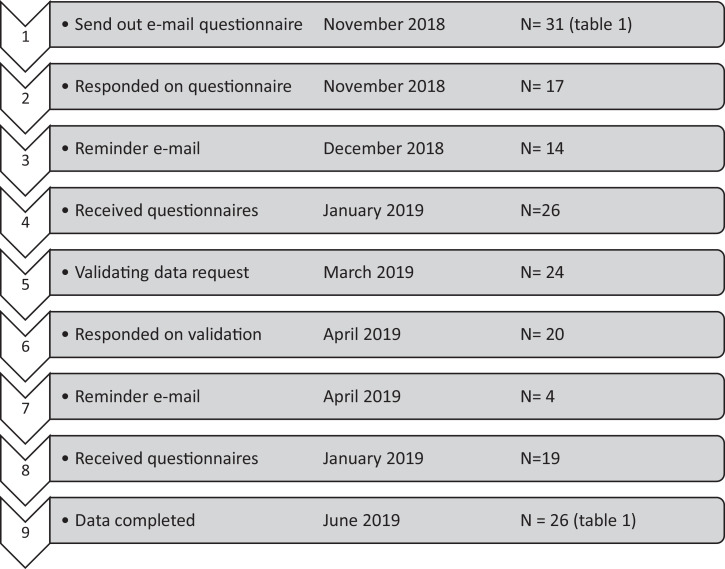
Procedure, time span, and results questionnaire.

### General questions

The first questions were generic to outline the scope of the professional dental hygiene practice by country. In all but one of the countries, the DH is an officially recognised dental care professional. Only Austria does not recognise the dental hygiene profession but is nevertheless a member of the IFDH and EDHF. In 58% of the 26 countries, the DH is allowed to work independently, without supervision by a dentist. Direct access to a DH is allowed in 54% of the countries. The highest dental hygiene education level for the majority of the countries is a diploma or bachelor's degree (Table 2).

**Table 2:** Overview per country regarding education level, recognition, independent practice, and direct access for the dental hygiene profession.

Country	Education level	Recognised	Independent practice	Direct access
Australia	Bachelor's degree, other	Yes	No	Yes
Austria	Other	No	No	No
Canada	Diploma, bachelor's degree	Yes	Yes	Yes
Czech Republic	Bachelor's degree, diploma	Yes	Yes	No
Denmark	Bachelor's degree	Yes	Yes	Yes
Finland	Bachelor's degree	Yes	Yes	Yes
Ireland	Diploma	Yes	No	No
Israel	Diploma	Yes	No	No
Italy	Bachelor's degree	Yes	Yes	No
Japan	Associate/bachelor's degree	Yes	No	No
Latvia	Diploma	Yes	Yes	Yes
Lithuania	Associate/bachelor's degree	Yes	Yes	Yes
Malta	Bachelor's degree	Yes	No	No
Netherlands	Bachelor's degree	Yes	Yes	Yes
Norway	Bachelor's degree	Yes	Yes	Yes
Poland	Diploma/associate degree	Yes	No	No
Portugal	Bachelor's degree	Yes	No	Yes
Russia	Diploma	Yes	No	No
Singapore	Diploma	Yes	Yes	No
Slovakia	Bachelor's degree	Yes	Yes	No
South Africa	Bachelor's degree	Yes	Yes	Yes
Spain	Other	Yes	No	No
Sweden	Bachelor's degree	Yes	Yes	Yes
Switzerland	Diploma	Yes	Yes	Yes
UK	Diploma/bachelor's degree	Yes	Yes	Yes
USA	Associate degree	Yes	No	Yes
**Percentage**		**25/26** **96%**	**15/26** **58%**	**14/26** **54%**

### Radiology

In 77% of the countries, DHs are allowed to take radiographs, whereas in 6 countries (ie, Austria, Czech Republic, Italy, Japan, Russia, and Slovakia), DHs are not allowed to take radiographs. Of those who can take a radiograph, 45% do not need a referral, whereas 30% require a referral from a dentist. It remains unclear whether those in Latvia, Poland, Singapore, Spain, and Poland require a referral from a dentist. In 42% of the countries, the DH can indicate a radiograph and in 50% the DH is allowed to make a diagnosis from a radiograph. However, in only 31% of the countries a DH can do both. (Table 3).

**Table 3:** Overview per country regarding radiology aspects such as owning equipment, needed referral, indicating, taking, diagnosing, and type of radiographs for the dental hygiene profession.

Country	Own radiographic equipment	Indicate radiographs	Referral	Take radiographs	Type of radiographs	Diagnose from radiographs
Australia	No	Yes	No	Yes	IO-PAN-CT	CAR-PER
Austria	No	No	No	No	None	No
Canada	Yes	Yes	No	Yes	IO-PAN	O
Czech Republic	No	No	No	No	None	No
Denmark	Yes	Yes	No	Yes	IO-PAN	CAR-PER
Finland	No	No	Yes	Yes	IO-PAN	No
Ireland	No	No	Yes	Yes	IO-PAN	No
Israel	No	Yes	No	Yes	IO	PER
Italy	No	No	No	No	None	No
Japan	No	No	No	No	None	No
Latvia	No	No	No	Yes	IO-PAN	No
Lithuania	Yes	No	?	Yes	IO-PAN	No
Malta	No	No	Yes	Yes	IO-PAN	PER
Netherlands	No	No	Yes	Yes	IO-PAN	CAR-PER
Norway	Yes	Yes	No	Yes	IO-PAN	CAR-PER
Poland	No	No	?	Yes	PAN	CAR-PER
Portugal	No	Yes	Yes	Yes	IO-PAN	CAR-PER-O
Russia	No	No	Yes	No	None	No
Singapore	No	No	?	Yes	IO-PAN	No
Slovakia	No	No	No	No	None	PER
South Africa	Yes	Yes	?	Yes	IO-PAN-CT	CAR-PER
Spain	No	No	?	Yes	IO-PAN-CT	No
Sweden	Yes	Yes	No	Yes	IO	CAR-PER
Switzerland	No	Yes	Yes	Yes	IO-PAN-CT	CAR-PER
UK	No	Yes	No	Yes	IO-PAN	CAR-PER
USA	Yes	Yes	No	Yes	IO-PAN-CT	No
**Percentage**	**27%**	**58%**	**19%**	**77%**	**IO: 73%** ***PAN: 69%*** **CT: 19%**	**54%**

IO, intra-oral radiographs; PAN, panoramic radiographs; CT, cone beam CT scan; CAR, cariology; PER, periodontology and bone level; O, other; ?, unknown.

**Table 4:** Survey data per country and question.

Country	Recognised	Independent Practice	Direct Access	Education Level	Own Rad. Equipment	Indicate Radiographs	Referral	Take Radiographs	Type of Radiographs	Diagnose from Radiographs
Australia	Yes	No	Yes	Bachelor's degree, other	No	Yes	No	Yes	IO-PAN-CB	CAR-PER
Austria	No	No	No	Other	No	No	No	No	None	No
Canada	Yes	Yes	Yes	Diploma, bachelor's degree	Yes	Yes	No	Yes	IO-PAN	O
Czech Republic	Yes	Yes	No	Bachelor's degree, diploma	No	No	No	No	None	No
Denmark	Yes	Yes	Yes	Bachelor's degree	Yes	Yes	No	Yes	IO-PAN	CAR-PER
Finland	Yes	Yes	Yes	Bachelor's degree	No	No	Yes	Yes	IO-PAN	No
Ireland	Yes	No	No	Diploma	No	No	Yes	Yes	IO-PAN	No
Israel	Yes	No	No	Diploma	No	Yes	No	Yes	IO	PER
Italy	Yes	Yes	No	Bachelor's degree	No	No	No	No	None	No
Japan	Yes	No	No	Associate/bachelor's degree	No	No	No	No	None	No
Latvia	Yes	Yes	Yes	Diploma	No	No	No	Yes	IO-PAN	No
Lithuania	Yes	Yes	Yes	Associate/bachelor's degree	Yes	No	-	Yes	IO-PAN	No
Malta	Yes	No	No	Bachelor's degree	No	No	Yes	Yes	IO-PAN	PER
Netherlands	Yes	Yes	Yes	Bachelor's degree	No	No	Yes	Yes	IO-PAN	CAR-PER-O
Norway	Yes	Yes	Yes	Bachelor's degree	Yes	Yes	No	Yes	IO-PAN	CAR-PER
Poland	Yes	No	No	Diploma/associate degree	No	No	-	Yes	PAN	CAR-PER
Portugal	Yes	No	Yes	Bachelor's degree	No	Yes	Yes	Yes	IO-PAN	CAR-PER-O
Russia	Yes	No	No	Diploma	No	No	Yes	No	None	No
Singapore	Yes	Yes	No	Diploma	No	No	-	Yes	IO-PAN	No
Slovakia	Yes	Yes	No	Bachelor's degree	No	No	No	No	None	PER
South Africa	Yes	Yes	Yes	Bachelor's degree	Yes	Yes	-	Yes	IO-PAN-CT	CAR-PER
Spain	Yes	No	No	Other	No	No	-	Yes	IO-PAN-CT	No
Sweden	Yes	Yes	Yes	Bachelor's degree	Yes	Yes	No	Yes	IO	CAR-PER
Switzerland	Yes	Yes	Yes	Diploma	No	Yes	Yes	Yes	IO-PAN-CT	CAR-PER
UK	Yes	Yes	Yes	Diploma/bachelor's degree	No	Yes	No	Yes	IO-PAN	CAR-PER
USA	Yes	No	Yes	Associate degree	Yes	Yes	No	Yes	IO-PAN-CT	No

IO, intra-oral radiographs; PAN, panoramic radiographs; CT, cone beam ct-scan; CAR, cariology; PER, periodontology and bone level; O, other.

Concerning extraoral radiographs, Sweden and Israel do not allow DHs to take panoramic radiographs, which is in contrast to Poland, where only panoramic radiographs are allowed to be taken. In Australia, South Africa, Spain, Switzerland, and the US, the DH can take Cone Beam Computer Tomografie-scans scans.

In the 27% of the countries where a DH can legally own radiographic equipment, DHs can also provide the indication and take radiographs, except for in Lithuania. A DH can take radiographs but is not allowed to provide the indication in the following countries: Finland, Ireland, Latvia, Lithuania, Malta, the Netherlands, Poland, Singapore, and Spain. Although DHs can take radiographs, they may not make a diagnosis in Finland, Latvia, Ireland, Lithuania, Spain, Singapore, or the US.

In 5 countries (19%) the full set of tasks and skills is allowed, meaning that the DH may own radiographic equipment, indicate as well as take radiographs, and make a diagnosis with the help of radiographs.

## Discussion

This study aimed to summarise the scope of practice of the DH regarding oral radiology through a worldwide review. It provides information regarding the professional landscape of the DH and explores the research field.^11^ A questionnaire is an economical and efficient method of collecting data, but it needs to be carefully designed to ensure that the standardised questions provide uniform data to be recorded to increase the reliability of the study.^10^, ^11^, ^12^ This was established by performing a pilot study. The results of the final questionnaire show that qualifications, skills, and scope of practice regarding oral radiology vary by country.

In the present study, a response rate of 84% of the associations was obtained. The response rate could have been higher if all associations provided the IFDH website with accurate contact details. The reasons for nonresponse may be uncertainty regarding the researchers’ intentions, which are related to a dental school, and uncertainty concerning the purpose and how the study results would be used. However, nonresponse occurred despite the support by the IFDH, EDHF, and NVM, which was aimed to build confidence and trust. Privacy-related issues may have been another aspect of concern, although all associations were informed that identification of responders would not be possible, as the data were gathered anonymously. Nonresponse may also have been due to a language barrier. Scientific evidence indicates that the language of a questionnaire may affect the way international respondents provide answers to the same question.^13^^,^^14^ The researchers asked the responders a second time to verify the understanding of the data to ensure the correct interpretation and representation of their answers. The response rate for this validation email was 79%.

Five countries did not respond to the questionnaire, even though they were contacted through the publicly available email addresses. Nepal was one of those countries, which was remarkable as several papers have addressed the field of dental hygiene and the professional profile in this country.^15^^,^^16^ One of the reasons for this may be that the dental hygiene profession is relatively new to Nepal. Moreover, the DH professional's identity is not yet established, and the profession is not recognised. Consequently, a nationally agreed scope of practice for DHs and practice regulation is currently absent.^16^ The German association informed the research team that they decided not to complete the questionnaire because the dental hygiene profession is still under development and does not have legal status. Therefore, Germany was considered a nonresponder. However, Austria did complete the questionnaire regardless of the fact of the situation effectively similar to Germany, where the dental hygiene profession is currently not recognised.^17^

Worldwide, no homogenous legal position exists regarding radiology performed by a DH. Legislation of the DH profession in countries such as Australia, Canada, Switzerland, and the US is multi-jurisdictional and has a regional basis.^2^ Differences in the scope of practice of the DH are unique in each of the 50 states of the US. For a review of practice by state, the American Dental Hygiene Association publishes documents on their website^18^^,^^19^ Differences also apply to Canada, where the DH profession is regulated by province, as mentioned by the responder.^20^ The data from these countries are summarised and presented as the common minimum tasks allowed in most states and provinces. Furthermore, there is diversity in when the professional title DH can be used, based on diploma programmes with the duration of not even 2 years up to a 4-year bachelor's degree.

Another complicating factor is the recognition of the dental therapist (DT) profession. This profession currently exists in the US, New Zealand, Australia, Canada, and the UK.^21^ The development of the DT in the US grew from a desire to find a workforce solution for the increasing demand for oral health care. DTs are educated to the same standards of care as a dentist for their defined scope of practice and currently provide care under the supervision of a licensed dentist through a collaborative management agreement or standing orders.^21^ In the US, DHs do not have the same scope of practice as DTs and vice versa.^22^ In some countries, the professions DH and DT are incorporated in one training and profession, for instance, the Netherlands. The present study focused specifically on the DH because it is the most common dental auxiliary profession worldwide and is organised by an international association structure.

Little is known about the DH and their (independent) use of ionising radiation in practice. A recently published consensus statement on a common European curriculum for DH reports that three European countries permit DHs to indicate radiographs.^23^ This statement was based on an overview of the regulatory framework in the health services sector by the European Commission.^24^

In addition, the recently published common European Curriculum for Dental Hygiene–Domain III: Patient-centred Care specifically mentioned oral radiology as area of competence. This comprising diagnostic radiography, including hazards and regulations relating to its use indication, taking and interpretation of the radiograph as well as radiographic assessment of the extra-oral and intra-oral soft and hard tissues of the orofacial region where appropriate.^25^ Additional studies reveal that DHs assess and diagnose with the help of radiographs, most often intraoral radiographs.^7^^,^^23^^,^^26^^,^^27^ The British Society of Dental and Maxillofacial Radiology considers that occlusal, dental panoramic, and skull views should remain outside the scope of practice of DHs.^28^ This is in accordance with the most recent developments in the Netherlands. The selected group of DHs with a bachelor's degree and adjunct trained competencies no longer are in need of a dentist's referral to take intra-oral radiographs and thus can indicate these themselves. The oversight of a dentist is still needed for taking extra-oral radiographs.^6^

Previous studies have been performed on attitudes towards the DHs’ extended scope of practice.^29^^,^^20^ The present study solely and specifically focused on the legal aspects of the profession. This paper is one of a series and explicitly emphasises radiology. Moreover, it is acknowledged that the DH has a large variety of competencies in the field of prevention and periodontology.^2^

### Limitations

The results of this study may differ from daily practice, as the illegal provision of oral care is a common problem^17^ that may be a symptom of the underlying health care system and social deficits, ranging from the lack of access to care and health inequities to problems of governance and law enforcement.^30^ In addition, in some countries, no legal regulations exist regarding DHs taking radiographs. Furthermore, countries with large populations in Asia and South America are not represented in the current data because they were not affiliated with the International Dental Hygiene Association and therefore did not meet the inclusion criteria. Also, in some countries the dental hygiene profession does not exist.

### Recommendations

Variation is apparent amongst the countries included in this research and sometimes even within these countries. Moreover, there is a continuous development in education programmes, the dental care work arena, and the legal perspectives. It is recommended to align the legal position and minimum competencies worldwide, which would make the mobility of the DH across nations also easier, without the need for compensation measures. Therefore, the development of a worldwide common framework is considered as a first step, as well as further research on the dental hygiene profession in countries that were not included in this study. A recently published statement by the EDHF contributed to this cause by establishing a common European curriculum.^23^

## Conclusions

This paper summarised the competencies and legal position of the DH regarding oral radiology through a worldwide review. The required qualifications, skills, and scope of practice of the DH regarding oral radiology vary by country and, within some countries, even vary by state or province.

## Author contributions

MB contributed to conception and design, acquisition of data, and analysis and interpretation of data and drafted the manuscript.

EB contributed to conception and design, analysis, and interpretation and critically revised the manuscript.

FvdW contributed to design, analysis, and interpretation and critically revised the manuscript.

DES contributed to conception and design, acquisition of data, and analysis and interpretation of data and critically revised the manuscript.

All authors gave final approval and agreed to be accountable for all aspects of work ensuring integrity and accuracy.

## Ethics approval

Institutional Review and Ethics Board of ACTA, reference code 201913.

## Funding

This research received no specific grant from any funding agency in the public, commercial, or nonprofit sectors. The work for this paper was funded by the regular academic appointments of the authors at the Academic Centre for Dentistry Amsterdam (ACTA).

## Conflicts of interest

The authors declare that they have no conflict of interest.

## Appendix Supplementary materials

**Supporting information:** Additional supporting information may be found online in the supporting information section. Image, application 1
